# A Place for Us: A Unique Experience That Provides a Creative and Social Experience for People With Dementia

**DOI:** 10.1093/geroni/igaa057.2349

**Published:** 2020-12-16

**Authors:** Mary Mittelman, Amy Harris

**Affiliations:** 1 NYU School of Medicine, New York, New York, United States; 2 NYU School of Medicine, NEW YORK, New York, United States

## Abstract

A Place for Us meets weekly in community locations for a half day and is run by a recreation therapist who is also an artist, offering an opportunity to connect with others through participation in creative projects. We have found that collaborating in creating a piece of art, such as a collage provides a context for socializing. Since the program began in 2017, we provided this opportunity to 83 caregivers. The program has broad appeal and has included both men (54%) and women (46%) and people from many racial and ethnic backgrounds: 62.3% white, 13 percent African American, 7% Latino and 5% Asian. Our experience suggests that Interacting through collaborative projects utilizing what has been called, “The creative brain,” offers people with dementia an opportunity to feel at ease in this social setting. A Place for Us allows people to interact socially while engaging in a pleasant and normative activity.

